# Primitive and definitive erythropoiesis in mammals

**DOI:** 10.3389/fphys.2014.00003

**Published:** 2014-01-28

**Authors:** James Palis

**Affiliations:** Department of Pediatrics, Center for Pediatric Biomedical Research, University of Rochester Medical CenterRochester, NY, USA

**Keywords:** primitive erythropoiesis, definitive erythropoiesis, yolk sac, globin, cytoskeleton

## Abstract

Red blood cells (RBCs), which constitute the most abundant cell type in the body, come in two distinct flavors- primitive and definitive. Definitive RBCs in mammals circulate as smaller, anucleate cells during fetal and postnatal life, while primitive RBCs circulate transiently in the early embryo as large, nucleated cells before ultimately enucleating. Both cell types are formed from lineage-committed progenitors that generate a series of morphologically identifiable precursors that enucleate to form mature RBCs. While definitive erythroid precursors mature extravascularly in the fetal liver and postnatal marrow in association with macrophage cells, primitive erythroid precursors mature as a semi-synchronous cohort in the embryonic bloodstream. While the cytoskeletal network is critical for the maintenance of cell shape and the deformability of definitive RBCs, little is known about the components and function of the cytoskeleton in primitive erythroblasts. Erythropoietin (EPO) is a critical regulator of late-stage definitive, but not primitive, erythroid progenitor survival. However, recent studies indicate that EPO regulates multiple aspects of terminal maturation of primitive murine and human erythroid precursors, including cell survival, proliferation, and the rate of terminal maturation. Primitive and definitive erythropoiesis share central transcriptional regulators, including Gata1 and Klf1, but are also characterized by the differential expression and function of other regulators, including myb, Sox6, and Bcl11A. Flow cytometry-based methodologies, developed to purify murine and human stage-specific erythroid precursors, have enabled comparative global gene expression studies and are providing new insights into the biology of erythroid maturation.

## Introduction

RBCs comprise the most abundant cell type in the body and function primarily to transport oxygen and carbon dioxide. More than a century ago it was recognized that the mature RBCs in the bloodstream of mammals lack a nucleus, while the RBCs of adult birds, amphibians, and fish retain a nucleus throughout their lifespan in the circulation (Gulliver, 1875). Examination of the bloodstream of developing mammalian embryos at the beginning of the last century revealed the presence of two distinct, temporally overlapping populations of erythroid cells. The earliest population consisted of larger, nucleated cells and the subsequent population consisted of smaller, enucleated RBCs (Maximow, 1909). The transient presence of the larger, nucleated erythroid cells during early development was termed “primitive” erythropoiesis to distinguish it from the “definitive” form of erythropoiesis that occurred not only during late fetal life but throughout all of postnatal life. This review provides an overview of primitive and definitive erythropoiesis and relies primarily on studies of the mouse as a model of mammalian biology where these distinct erythroid lineages have been most intensively studied.

## Definitive erythropoiesis

**Figure 1:**
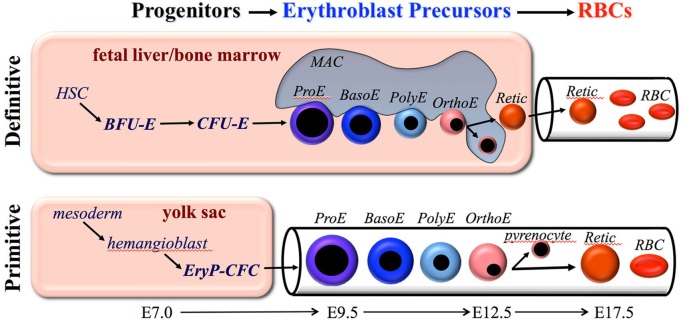
**Overview of primitive and definitive erythropoiesis**. Both forms of erythroid cell production are characterized by the progressive movement of cells through three compartments: progenitors, erythroblast precursors, and red blood cells (RBCs). Erythroid progenitors (BFU-E, CFU-E, and EryP-CFC) are defined by their capacity to form colonies of maturing erythroid cells *in vitro*. Erythroid precursors are defined morphologically as proerythroblasts (ProE), basophilic erythroblasts (Baso), polychromatophilic erythroblasts (PolyE), and orthochromatic erythroblasts (OrthoE). OrthoE enucleate to form a pyrenocyte, that contains the condensed nucleus, and a reticulocyte (Retic), that goes on to mature into a RBC. Definitive erythropoiesis in the adult organism is derived from hematopoietic stem cells (HSC), while primitive erythropoiesis occurs just once from mesoderm cells in the early embryo.

Definitive erythropoiesis occurs in the fetal liver and postnatal bone marrow and is characterized by the movement of lineage-committed cells through progenitor, precursor, and mature RBC compartments (Figure 1). The progenitor and precursor compartments occur in protected extravascular spaces and are associated with cellular amplification and maturation. These compartments sustain the third, functional compartment, which consists of RBCs circulating within the vascular network.

### Definitive erythroid progenitors

The most immature erythroid compartment consists of lineage-committed, definitive erythroid progenitors, termed burst-forming unit erythroid (BFU-E) and colony-forming unit erythroid (CFU-E). These progenitors are defined by their ability to form colonies of mature erythroid cells in semisolid media. BFU-E-derived colonies require 7 and 14 days in mouse and human systems, respectively, to form mature colonies typically containing more than a thousand erythroid cells. In contrast, the more mature CFU-E progenitors require only 2 and 7 days in mouse and human systems, respectively, to form mature colonies that consist of only 16–32 cells. Thus, CFU-E are only 4–5 cell divisions upstream of mature RBCs.

The cytokine erythropoietin (EPO) is necessary for the ability of CFU-E to generate colonies *in vitro*. CFU-E are exquisitely dependent on EPO for their survival (Koury and Bondurant, 1990). EPO levels in the bloodstream, regulated by hypoxia, are thought to modulate the number of CFU-E in the marrow and thus regulate the output of definitive RBCs (Koury and Bondurant, 1992). EPO acts through its specific receptor, EPOR, characterized by a single transmembrane domain that dimerizes upon ligand activation. Downstream signals occur through Jak2/Stat5, PI3K/AKT, and MAPK pathways (reviewed by Richmond et al., 2005). The importance of EPO signaling through its cognate receptor was demonstrated by the identical phenotype of EPO-null and EPOR-null mice: fetal death at embryonic day 13.5 (E13.5) associated with severe anemia and the complete lack of definitive RBCs (Wu et al., 1995; Kieran et al., 1996; Lin et al., 1996). The restoration of EPOR specifically in erythroid lineages rescued the lethal phenotype, indicating that anemia is indeed the cause of *in utero* death (Suzuki et al., 2002). Recent studies provide evidence that BFU-E and CFU-E can undergo limited self-renewal, particularly in response to acute stress such as anemia and that the self-renewal of CFU-E is dependent on the action of EPO (Flygare et al., 2011; Peslak et al., 2012).

### Definitive erythroid precursors

The second erythroid compartment consists of morphologically identifiable, nucleated precursors that progress from proerythroblast (ProE) to basophilic (BasoE), polychromatophilic (PolyE), and orthochromatic (OrthoE) forms (Figure 1). Erythroid precursor maturation is characterized by progressive (1) erythroblast expansion through a limited set of symmetric cell divisions, (2) accumulation of hemoglobin, (3) decrease in cell size, (4) nuclear pyknosis, and (5) decrease in RNA content. The morphologic criteria distinguishing these cells has relied primarily on the progressive nuclear condensation combined with changes in cytoplasmic staining, which reflects the degree of hemoglobin and RNA content (Figure 1). Enhanced flow cytometric approaches to distinguish these subpopulations have been developed for both mouse and human systems and have provided the capacity to analyze large numbers of cells and to physically isolate these precursors for further study (Koulnis et al., 2011; Hu et al., 2013; Kingsley et al., 2013; Liu et al., 2013). In addition, an imaging flow cytometry-based approach, which weds immunophenotypic analyses with characterization of morphologic features, has been developed to quantify multiple cellular aspects associated with progressive erythroid maturation, including their enucleation (McGrath et al., 2008a; Peslak et al., 2011, 2012; Konstantinidis et al., 2012).

Definitive erythroblasts mature in the fetal liver and postnatal bone marrow within erythroblastic islands, composed of erythroblasts physically attached to central macrophage cells (reviewed by Chasis and Mohandas, 2008). Macrophages have been shown to promote erythroblast proliferation, particularly in the context of stress erythropoiesis (Hanspal et al., 1998; Rhodes et al., 2008; Chow et al., 2013; Ramos et al., 2013). In mammals, the end result of precursor maturation is enucleation, which results in the formation of two cell types. The first population consists of reticulocytes that contain most of the cytoplasm and hemoglobin, as well as the proteins needed to form a unique cytoskeletal network (Gaiduschek and Singer, 1979; Koury et al., 1989; Lee et al., 2004; Liu et al., 2011). The second population consists of pyrenocytes (“extruded nuclei”) that contain the condensed nucleus surrounded by a lipid bilayer and thin rim of cytoplasm (McGrath et al., 2008a). Enucleation is a complex process that involves multiple steps including the establishment of cell polarity through microtubule action, the formation of a contractile actomyosin ring, vesicle formation, and coalescence of lipid rafts between reticulocyte and pyrenocytes (Keerthivasan et al., 2010; Konstantinidis et al., 2012; Wang et al., 2012). Soon after their formation in the fetal liver and bone marrow, pyrenocytes flip phosphotidylserine onto their cell surface, providing an “eat me” signal, and are rapidly ingested by macrophages (Yoshida et al., 2005).

Reticulocyte maturation is a complex process that results in an approximately 20% loss of plasma membrane surface area, reduced cell volume, increased association of the cytoskeleton to the outer plasma membrane, and the loss of all residual cytoplasmic organelles, including mitochondria and ribosomes (Johnstone, 1992; Waugh et al., 2001). Organelle clearance occurs through both autophagy and exocytosis (Griffiths et al., 2012). The membrane changes convert a multilobulated immature reticulocyte into a biconcave disc with improved viscoelasticity. All of these changes prepare the reticulocyte for its rigorous sojourn of 45 and 120 days in the bloodstream of adult mice and humans, respectively.

### Definitive RBCs

The third erythroid compartment consists of circulating reticulocytes and mature RBCs (Figure 1). Steady-state levels of RBCs are maintained by the continuous production and release of reticulocytes into the bloodstream to balance the removal of senescent RBCs by macrophage cells, which are localized primarily in the spleen (Bennett and Kay, 1981). In adult humans, this steady-state production results in the egress of more than 2 million reticulocytes every second into the bloodstream. This incredible number is estimated as follows: adult humans normally contain approximately 5 × 10^6^ RBCs per microliter of blood and the blood volume of a 70 kg person is approximately 5 l, which amounts to 2.5 × 10^13^ total circulating RBCs. Since the lifespan of human RBCs is approximately 115 days (Franco, 2012), we replace on average 1/115th of our red cells every day, or 2.2 × 10^11^ RBC/day, which amounts to 2.5 × 10^6^ RBCs/s.

The membrane of mature definitive RBCs is composed of a cholesterol/phospholipid plasma membrane to which is anchored an elastic network of spectrin-based cytoskeletal proteins containing actin and protein 4.1 at their junctions (reviewed by Mohandas and Gallagher, 2008). The anchoring of the cytoskeleton to the lipid bilayer occurs at these junctions through glycophorin C. In addition, interactions through ankyrin-band 3 stabilize the cytoskeleton to the lipid bilayer. This unique cytoskeletal network ensures that mature RBCs can passively deform during their repeated passage through capillary networks, yet ultimately maintain their biconcave shape. All of the components of the cytoskeleton accumulate during erythroblast maturation (Chen et al., 2009). Abnormalities of cytoskeletal proteins lead to hemolytic disorders in children and adults, including hereditary spherocytosis and elliptocytosis (Gallagher, 2013).

As RBCs age, surface area and volume, but not hemoglobin, are progressively lost resulting in increased RBC density. Senescent RBCs are cleared by splenic macrophages that recognize, among other changes, clustered band 3 on the RBC surface (Low et al., 1985). In addition, CD47 interactions with thrombospondin-1 on senescent RBCs can convert “don't eat me” to “eat me” signals leading to RBC clearance by macrophage cells (Burger et al., 2012).

## Primitive erythropoiesis

### Primitive erythropoiesis- emergence in the yolk sac from a transient wave of progenitors

The first blood cells in the mammalian conceptus emerge in “blood islands” within the yolk sac. These pools of primitive erythroblasts differentiate from mesoderm cells soon after the onset of gastrulation. Examination of carefully staged mouse embryos revealed the presence of a unique erythroid progenitor, termed EryP-CFC (Wong et al., 1986; Palis et al., 1999). This transient wave of EryP-CFC emerges in the yolk sac at E7.25, peaks in numbers at E8.25, and are no longer detectable at E9.0 (Palis et al., 1999). EryP-CFC, like their definitive erythroid progenitor counterparts, require the addition of EPO for *in vitro* colony formation. Murine EryP-CFC require 5 days of *in vitro* culture to form colonies containing several hundred mature primitive erythroid cells. Thus, EryP-CFC contain a proliferative potential that is intermediate to that of the definitive BFU-E and CFU-E progenitors. In addition, primitive erythroid progenitors, unlike definitive erythroid progenitors, are incapable of even limited self-renewal when cultured *ex vivo* in the presence of EPO, SCF and dexamethasone, a difference associated with the differential expression of the receptors for the latter two factors (England et al., 2011).

A global gene expression study of E7.5 and E8.5 primitive erythroid cells reveals a gene profile characteristic of high aerobic glycolysis, suggesting that these progenitors share the “Warburg effect” with many cancer cell types that are found in hypoxic environments (Isern et al., 2011). Consistent with a role for hypoxia signaling in EryP-CFC, increases in progenitor numbers and in colony size were detected when yolk sac cells were cultured in low oxygen compared with room air. Interestingly, low oxygen conditions facilitated the detection of BFU-E in early mouse embryos, suggesting that hypoxia signaling also plays a role in the emergence of definitive erythroid progenitors in the yolk sac (Borges et al., 2012).

### Primitive erythropoiesis- terminal precursor maturation

Examination of circulating blood cells over developmental time in rodent embryos revealed that primitive erythroblasts undergo progressive morphological maturation (Morioka and Minamikawa-Tachino, 1993; Kingsley et al., 2004; Fraser et al., 2007). Consistent with their origin from a transient wave of yolk sac-derived EryP-CFC at E7.5–E8.5, a semi-synchronous wave of maturing nucleated primitive erythroid precursors is evident in the mouse embryo between E9.5–E12.5 (Figure 1). Primitive erythroid precursors mature with progressive characteristics similar to their definitive counterparts, including (1) expansion of erythroblast numbers through a limited set of symmetric cell divisions, (2) accumulation of hemoglobin, (3) decrease in cell size, (4) nuclear pyknosis, and (5) decrease in RNA content. In addition, primitive erythroblasts lose the intermediate filament vimentin and nuclear histone proteins, changes that also occur during definitive erythroblast maturation (Sangiorgi et al., 1990; Morioka et al., 1998). By E12.5, the primitive erythroblasts have matured to an orthochromatic stage and cell division ceases.

The unique expression in primitive erythroid cells of embryonic globin genes both from the beta and from the alpha globin clusters has facilitated their identification and study, even after definitive cells have begun to emerge in the fetus. From the beta globin cluster, the embryonic εy- and βH1-globin genes are expressed in the mouse, and the ε- and γ-globin genes are expressed in human (Table 1). In contrast, the “adult” β1- and β2-globin genes are expressed in the mouse, and “fetal” γ- and “adult” β-globin genes are expressed in human definitive erythroid cells. From the alpha globin cluster in both mouse and human, primitive cells also express the embryonic ζ- and α-globin genes. The change in the expression of embryonic, fetal (for human), and adult globin genes during development has been termed “switching” and has been extensively studied over the past several decades with the ultimate goal of treating children and adults with hemoglobinopathies (reviewed by Stamatoyannopoulos, 2005; Sankaran et al., 2010). The transcriptional regulators Sox6 and Bcl11A have been identified as important regulators of globin gene expression, since they are expressed in definitive, but not primitive, erythroid cells, where they act to suppress the expression of embryonic globin genes (Yi et al., 2006; Sankaran et al., 2009). Erythroid cells can also switch their hemoglobin expression as they mature. This has been best characterized in primary mouse primitive erythroblasts, which undergo βH1- to εy-globin and ζ- to α-globin “maturational” globin switches (Kingsley et al., 2006).

**Table 1:** **Expression of globin genes and transcriptional regulators in primitive and definitive murine erythroid cells**.

	**Primitive erythropoiesis**	**Definitive erythropoiesis**
Globin gene expression	ζ and α, βH1 and εy >> β1 and β2	α, almost exclusively β1 and β2
Transcriptional regulators	Gata1, Klf1, Tal1, Lmo2, Ldb1	Gata1, Klf1, Tal1, Lmo2, Ldb1, Myb, Sox6, Bcl11a

For more than a century it was thought that primitive erythroblasts remain nucleated throughout their lifespan in the fetal circulation. However, large, enucleated “megalocytes” having the same hemoglobin content as nucleated yolk sac erythroblasts were detected at late stages of gestation in the mouse embryo (Bethlenfalvay and Block, 1970; Steiner and Vogel, 1973). Using antibodies specific for embryonic globins to unequivocally identify primitive erythroid cells, it was determined that primitive erythroblasts in the mouse fetus enucleate between E12.5–E16.5 of gestation (Kingsley et al., 2004). These findings have been confirmed in mice containing a GFP transgene driven by the human ε-globin promoter to identify primitive erythroid cells (Fraser et al., 2007). A transient population of primitive pyrenocytes was also detected during this time period in the fetal circulation of mice (McGrath et al., 2008b). Importantly, the total number of primitive erythroid cells does not decrease between E12.5 and E16.5, consistent with the cessation of cell division in late-stage primitive erythroblasts and the enucleation of the entire population of primitive erythroid cells (Kingsley et al., 2004).

### Primitive RBCs

Reticulocyte maturation is associated with a significant loss of surface area and volume associated with remodeling of the cytoskeleton. Extremely little is known about the components of membrane cytoskeleton of primitive erythroid cells. The expression of band 3 and glycophorin A are highly upregulated as early as E8.5 (Isern et al., 2011). Spectrin and ankyrin transcripts have been identified in E10.5–E12.5 primitive erythroblasts (Peters et al., 1992). Since primitive erythroblasts mature in the circulation, they may assemble a functional cytoskeleton prior to their enucleation and terminal maturation into RBCs. However, it is not known when during their maturation the cytoskeleton is assembled. We have recently examined the biomechanical properties of murine primitive erythroid cells during the developmental time they undergo enucleation, i.e., between E12.5 and E17.5 (Waugh et al., 2013). Late-stage primitive erythroblasts have membrane deformability similar to mature definitive RBCs, a finding consistent with their need to survive the stresses of fetal circulation. As primitive erythroid cells enucleate between E12.5 and E17.5, the physical association of their outer membrane bilayer with the underlying cytoskeleton increases. Primitive erythroid cells also lose 35% of their surface area and 50% of their volume between E14.5 and E17.5. Interestingly, the loss of surface area and volume occurs whether or not the cells are enucleated (Waugh et al., 2013). These data suggest that, unlike definitive erythropoiesis, the maturational processes of membrane remodeling and enucleation are uncoupled in terminally maturing primitive erythroid cells.

While the lifespan of primitive RBCs is not known, primitive RBCs have been detected for several days after birth (Kingsley et al., 2004; Fraser et al., 2007). These data indicate that primitive RBCs can circulate for at least 5–7 days after they enucleate. It is not known where or by what mechanisms senescent primitive RBCs are recognized and cleared from the vasculature.

### The role of EPO in primitive erythropoiesis

The role of EPO in primitive erythropoiesis has been surrounded by controversy. Early studies showed that EPO failed to increase heme synthesis in cultures of whole mouse embryos containing primitive erythroid cells (Cole and Paul, 1966), but the same group subsequently reported increased heme synthesis when EPO was added to cultures of disaggregated embryonic cells (Bateman and Cole, 1971). EPOR transcript expression has been identified in yolk sac blood islands between E7.5–E8.5 and in the yolk sac of E9.6–11.5 mouse embryos (McGann et al., 1997; Makita et al., 2001), while EPOR protein has been quantified on the cell surface of maturing primitive erythroblasts in the fetal hamster (Boussios et al., 1988). Furthermore, EPO increased erythroid cell numbers and embryonic globin expression in E7.5 yolk sac explants (Palis et al., 1995). Targeted disruption of EPOR causes a marked reduction of primitive erythroblasts by E10.5–E11.5 and a profound anemia by E12.5 (Wu et al., 1995; Kieran et al., 1996; Lin et al., 1996; Malik et al., 2013). Recently, a more detailed analysis of primitive erythropoiesis in EPOR-null mouse embryos has revealed important functions of EPOR signaling in terminal erythropoiesis, including reduction of primitive erythroblast proliferation associated with increased p27 expression, advanced cellular maturation, and markedly elevated rates of apoptosis associated with an imbalance in pro- and anti-apoptotic gene expression (Malik et al., 2013). Little EPO is expressed in the early mouse yolk sac (Malik et al., 2013), however, neuroepithelial cells of E8.5–E11.5 mouse embryos have recently been shown to express EPO and likely serve to support the early maturation of primitive erythroid precursors (Suzuki et al., 2013).

### Human primitive erythropoiesis

While primitive erythropoiesis has been most thoroughly investigated in the mouse model, relatively little is known about primitive erythropoiesis in humans owing both to ethical concerns and to the physical inaccessibility of the early embryo. Blood islands filled with primitive erythroblasts first arise at 18–20 days of gestation (Bloom and Bartelmez, 1940; Luckett, 1978; Kelemen et al., 1979). Primitive erythroblasts are the only circulating erythroid cells in human embryos from 3 to 6 weeks of gestation and nucleated primitive erythroid cells have been identified in the fetal circulation of the human embryo throughout the first trimester (Knoll, 1927). Human primitive erythroblasts can physically interact with macrophages within the placenta and, like their murine counterparts, also enucleate *in vivo* (Van Handel et al., 2010).

Human primitive erythroblasts express the embryonic ε- and ζ-globin genes. A maturational ζ- to α-globin switch occurs between 5 and 7 weeks of gestation in primitive erythroid cells (Peschle et al., 1985). Primitive erythroid cells derived from the *in vitro* differentiation of human ES cells can serve as a surrogate model system for early stages of human development. Studies of embryonic stem cell-derived human primitive erythroblasts indicate that they, like their primary counterparts, undergo a “maturational” switch from hemoglobin Gower I (ζ_2_ε_2_) to hemoglobin Gower II (α_2_ε_2_) (Qiu et al., 2008). Recently, primitive erythroblasts derived from human ES have been shown to require EPO for their terminal maturation. Similar to primitive erythropoiesis in EPOR-null embryos, human primitive erythroblasts deprived of EPO undergo increased apoptosis and accelerated maturation at late maturational stages (Malik et al., 2013). These studies indicate that the role of EPO in terminal maturation of primitive erythroid precursors is evolutionarily conserved in mammals.

Little is known about the cytoskeletal network of human primitive erythroid cells. However, a recent membrane proteomics analysis of primary human primitive erythroblasts reveals the presence of several cytoskeletal proteins, including spectrin, ankyrin, band 3, protein 4.1, and dematin, suggesting that the cytoskeleton of primitive and definitive erythroid lineages share common structural features (Ponnusamy et al., 2012).

## Ontogeny of primitive and definitive erythropoiesis

### The hemangioblast origin of primitive erythropoiesis

The emergence of primitive erythroid cells and a vascular network in close temporal and spatial proximity within the yolk sac suggested that these lineages might arise from common “hemangioblast” precursors (Sabin, 1920). This concept has been validated in the murine embryo using clonal assays, since cells containing both endothelial and hematopoietic potential have been localized to the primitive streak of gastrulating mouse embryos (Huber et al., 2004). Consistent with the emergence from hemangioblasts precursors, primitive erythroid potential has been derived from mesodermal cells expressing the endothelial/hematopoietic markers flk1, CD31 and Tie-2 (Ema et al., 2006). Studies of mouse and human embryonic stem cells differentiated *in vitro* toward blood cell fates also have provided evidence that hemangioblast precursors arise during gastrulation prior to the onset of hematopoietic progenitor activity (Choi et al., 1998; Zambidis et al., 2005; Kennedy et al., 2007).

### Definitive erythropoiesis- two developmental origins

Just as colony assays have been used to define the onset of primitive erythroid potential, a temporal and spatial analysis of definitive erythroid progenitors (BFU-E) in carefully staged mouse embryos reveals the onset of definitive erythroid potential within the yolk sac at E8.25, just prior to the onset of circulation (Palis et al., 1999). The emergence of BFU-E in the yolk sac is associated spatially and temporally with the emergence of multipotential hematopoietic progenitor cells (Palis et al., 2001), that have been termed “erythro-myeloid progenitors” (EMP). Definitive erythroid and multipotential hematopoietic progenitor activity has been generated *in vitro* from the culture of hemangioblasts, suggesting that EMP potential arises from hemangioblast precursors (Lacaud et al., 2002). Other evidence suggests that definitive hematopoietic potential emerges in the yolk sac from hemogenic endothelial cells composing part of the newly established vasculature(Li et al., 2005). More research is needed to clarify the developmental origins of definitive hematopoiesis in the mammalian embryo.

Following their initial emergence, BFU-E numbers expand rapidly in the yolk sac, are subsequently found in the bloodstream, and then in the early fetal liver (Palis et al., 1999). These yolk sac-derived definitive erythroid progenitors, when cultured *in vitro*, express predominantly adult β1- and β2-globins, but unlike their adult counterparts, they also express small amounts of the embryonic βH1-globin gene (McGrath et al., 2011). The first definitive RBCs are detected in the fetal bloodstream between E11.5 and E12.5 (McGrath et al., 2011). Mouse embryos lacking a heart beat and a functional circulation, contain similar numbers of BFU-E in the yolk sac but fail to distribute those BFU-E to the embryo proper, supporting the concept that the first definitive erythroid potential found in the fetal liver originates in the yolk sac (Lux et al., 2008). Interestingly, BFU-E in the human embryo are first detected in the yolk sac as early as 4–5 weeks gestation (Migliaccio et al., 1986). Similar to findings in the mouse, BFU-E are subsequently found in the fetal liver of the human embryo as soon as it begins to form as an organ (Migliaccio et al., 1986). These data indicate that definitive erythropoiesis first emerges after the onset of gastrulation in the yolk sac and seeds the early fetal liver.

In the adult, all blood cell lineages are derived from hematopoietic stem cells that are assayed by their ability to repopulate the entire blood system of lethally irradiated adult hosts. Using this functional assay, the first hematopoietic stem cells in the mouse embryo have been found to emerge, not from the yolk sac at E8.25, but from major arterial vessels, including the aorta, beginning at E10.5 (Kumaravelu et al., 2002). Hematopoietic stem cell activity subsequently expands in the fetal liver, peaking there at E16.5, and subsequently transitions to its long-term resident location in the bone marrow (Ema and Nakauchi, 2000; Wolber et al., 2002). This transition begins by E17.5 and 10–11 weeks in mouse and human embryos, respectively, but is not completed until after birth (Kelemen et al., 1979; Charbord et al., 1996; Blazsek et al., 2000).

Taken together, these studies support the conclusion that definitive erythropoiesis has two developmental origins. The first originates in the yolk sac of mouse and human embryos as a transient lineage that generates the first circulating definitive RBCs that emerge from the fetal liver. This transient definitive erythroid system is ultimately replaced by a second, permanent blood system, derived from hematopoietic stem cells, that provides for lifelong RBC production. However, further research is required to establish the timing of this transition from yolk sac-derived to hematopoietic stem cell-derived definitive erythropoiesis.

## Transcriptional regulation of primitive and definitive erythropoiesis

Transcription factors play critical roles driving lineage-specific cellular maturation. The Gata1 transcription factor plays a central role in the regulation of erythroid-specific genes both in primitive and in definitive erythroid cells. Disruption of Gata1 leads to the maturational arrest of both primitive and definitive erythroid lineages at the proerythroblast stage (Pevny et al., 1995; Fujiwara et al., 1996). Different functional domains of GATA1 are required to activate target genes in primitive vs. definitive erythroid cells (Shimizu et al., 2001), suggesting that different Gata1-containing transcriptional complexes may function in these lineages. Transcriptional regulators that can complex with Gata1 include Ldb1, FOG1, Scl/Tal1, and Lmo2. Targeted disruption of each of these genes leads to marked defects in primitive and definitive erythroid cells (Warren et al., 1994; Robb et al., 1995; Shivdasani et al., 1995; Tsang et al., 1998; Li et al., 2010).

Another important transcriptional regulator of erythropoiesis is the erythroid-specific Kruppel-like factor Klf1/EKLF (Miller and Bieker, 1993). Klf1 regulates the expression of several erythroid-specific genes including the adult and embryonic globins, alpha-hemoglobin stabilizing protein, heme biosynthetic enzymes, several transcription factors, as well as cytoskeletal proteins and blood group antigens that are expressed both in primitive and in definitive erythroid cells (Hodge et al., 2006; Nilson et al., 2006; Basu et al., 2007). Loss of even one allele of Klf1 significantly decreases the expression of Ter119 on the surface of primitive erythroblasts (Isern et al., 2010). Consistent with Klf1 regulation of multiple cytoskeletal genes, Klf1-null primitive erythroblasts display markedly abnormal cell membranes and ruffled cell surfaces.

While the primitive and definitive erythroid lineages share many transcription factors central to erythropoiesis, there are also several transcriptional regulators that are differentially expressed and function differentially in embryonic vs. fetal/adult erythroid lineages. Targeted disruption of the Myb gene completely blocks the maturation of definitive erythroblasts in the fetal liver but has no discernable effect in primitive erythropoiesis (Mucenski et al., 1991; Tober et al., 2008). Interestingly, Myb-null mouse embryos die of progressive anemia, but only after E15.5, indicating that the primitive erythroid lineage can support survival of the mouse fetus throughout most of its gestation. Humans with Trisomy 13 have persistence of embryonic and fetal hemoglobins associated with dysregulation of the Myb gene and upstream microRNas in fetal erythroblasts (Sankaran et al., 2011). Sox6 and Bcl11A are additional differentially expressed transcriptional regulators that down-regulate embryonic and fetal globin gene expression (Yi et al., 2006; Xu et al., 2010). Other transcription factors differentially expressed between primitive and definitive erythroid lineages have been identified through comparative bioinformatic analyses (Kingsley et al., 2013), however, functional studies confirming their differential expression and potential functions in the regulation of erythropoiesis have not been reported.

The complex interaction of transcription factors in the regulation of erythroid development has been modeled (Swiers et al., 2006). A summary of some of the key transcription factors regulating primitive and definitive erythropoiesis is provided in Table 1. In addition to the action of master erythroid transcription factors, it has recently been recognized that interferon regulatory factors, including IRF2 and IRF8, cooperate with Gata1 and Tal1, to regulate adult, but not fetal, human erythropoiesis (Xu et al., 2012). Interestingly, Irf8 is not expressed in primitive erythroblasts and IFNγ inhibits adult mouse definitive, but not primitive, erythroid colony formation (Greenfest-Allen et al., 2013). These data, taken together, suggest that inflammatory signals regulate adult, but not embryonic or fetal, erythropoiesis.

Several global gene expression analyses derived from primary primitive or definitive erythroid cells at various stages of maturation are available (Miller, 2004; Redmond et al., 2006; Isern et al., 2011; Merryweather-Clarke et al., 2011). Of note, a comparative analysis of global gene expression in similarly staged primitive, fetal definitive, and adult definitive erythroid cells has recently been published (Kingsley et al., 2013). A user-friendly website (http://www.cbil.upenn.edu/ErythronDB) has also been established, making these data readily available to the scientific community to facilitate comparative expression studies. The differential expression of globin genes, as well as several of their transcriptional regulators, have served as the primary genetic feature distinguishing primitive and definitive erythropoiesis (Table 1). In addition, several aquaporin gene family members were recently identified to be differentially expressed in primitive vs. adult definitive mouse erythroblasts (Kingsley et al., 2013). The expression of aquaporins 1 and 9 were confirmed in adult RBCs, while the specific expression of aquaporins 3 and 8 in primitive, but not adult definitive, erythroid cells correlates with their ability to accumulate reactive oxygen species when exposed to exogenous hydrogen peroxide. These studies raise the possibility that primitive erythroid cells may serve as a sink to protect the early embryo from free radical injury. Comparative global gene expression studies should continue to provide new insights into the biology of primitive and definitive erythroid maturation.

### Conflict of interest statement

The author declares that the research was conducted in the absence of any commercial or financial relationships that could be construed as a potential conflict of interest.
